# Genome-Wide Analysis of Effectors of Peroxisome Biogenesis

**DOI:** 10.1371/journal.pone.0011953

**Published:** 2010-08-04

**Authors:** Ramsey A. Saleem, Rose Long-O'Donnell, David J. Dilworth, Abraham M. Armstrong, Arvind P. Jamakhandi, Yakun Wan, Theo A. Knijnenburg, Antti Niemistö, John Boyle, Richard A. Rachubinski, Ilya Shmulevich, John D. Aitchison

**Affiliations:** 1 Institute for Systems Biology, Seattle, Washington, United States of America; 2 Department of Cell Biology, Faculty of Medicine & Dentistry, University of Alberta, Edmonton, Alberta, Canada; 3 Department of Signal Processing, Tampere University of Technology, Tampere, Finland; Baylor College of Medicine, United States of America

## Abstract

Peroxisomes are intracellular organelles that house a number of diverse metabolic processes, notably those required for β-oxidation of fatty acids. Peroxisomes biogenesis can be induced by the presence of peroxisome proliferators, including fatty acids, which activate complex cellular programs that underlie the induction process. Here, we used multi-parameter quantitative phenotype analyses of an arrayed mutant collection of yeast cells induced to proliferate peroxisomes, to establish a comprehensive inventory of genes required for peroxisome induction and function. The assays employed include growth in the presence of fatty acids, and confocal imaging and flow cytometry through the induction process. In addition to the classical phenotypes associated with loss of peroxisomal functions, these studies identified 169 genes required for robust signaling, transcription, normal peroxisomal development and morphologies, and transmission of peroxisomes to daughter cells. These gene products are localized throughout the cell, and many have indirect connections to peroxisome function. By integration with extant data sets, we present a total of 211 genes linked to peroxisome biogenesis and highlight the complex networks through which information flows during peroxisome biogenesis and function.

## Introduction

Peroxisomes are membrane-bound organelles that function in a variety of processes including the β-oxidation of long chain fatty acids and elimination of reactive oxygen species [1]. Disruption of the organelle has severe medical consequences; peroxisome biogenesis disorders are usually fatal in the first year of life. Peroxisomes are remarkably dynamic, responding to environmental and cellular cues by alterations in size, number and proteomic content. In the yeast *Saccharomyces cerevisiae*, peroxisomes proliferate when cells are incubated with fatty acids as the sole carbon source. Peroxisomal biogenesis results from the convergence of several processes including signaling [2], chromatin modifications [3] reorganization of the transcriptional networks [4], [5], [6], and the dynamics of the organellar proteome [7], [8].

Genome-wide studies using the ability of cells to grow on fatty acids as the primary phenotypic measure [9], [10], and measurements of the transcriptome in response to fatty acids [4], [5] have provided large data sets containing genes that are required for the metabolism of fatty acids or are responsive to the fatty acid-induced biogenesis. While different assays focused on identifying peroxisomal proteins can identify the same components, thereby reinforcing one another, distinct classes of proteins are also identified depending on the experimental approach or condition [7], [10]. For example in *S. cerevisiae* exposure to fatty acids dramatically induces the expression of genes encoding many peroxisomal proteins while concomitantly inducing the biogenesis and/or maturation of organelles; however, when compared to a fitness data set measuring growth of individual deletion strains on fatty acid-containing media, there is remarkably little overlap between the data sets [10].

A comprehensive understanding of the complex series of cellular events that occur in response to environmental stimuli requires both knowledge of the program executed and a full inventory of the players involved in its execution. We sought to determine in a genome-wide manner which genes are required for the normal establishment and maintenance of peroxisomes and to gain understanding of the underlying biological defects of deletions of many of these genes - both newly identified and those originally identified in other studies. By analyzing the resulting peroxisomes, we were able to establish subsets of defects that include underdeveloped peroxisomes, enlarged peroxisomes, an inability to express a peroxisomal reporter and peroxisome inheritance defects. We also integrate this study with additional datasets from the literature to develop a global picture of effectors of peroxisome biogenesis.

## Results

### Evaluation of Candidates by Flow Cytometry

A fully functional GFP-tagged chimera of the protein Pot1p, a thiolase localized to the peroxisomal matrix, was introduced into an arrayed library containing the complete collection of viable yeast deletion mutant strains (∼4000 strains after quality control selection - see Materials and Methods). To gain an initial assessment of each strain's ability to produce Pot1p-GFP (requiring transcription, translation, protein folding and/or stability) cells were subjected to flow cytometry at 16 hours after transfer from glucose to oleate (Table S1-1). From this analysis prioritized list of 186 candidates were assayed at early (6 hours) and late (24 hours) time points of induction.

**Figure 1:**
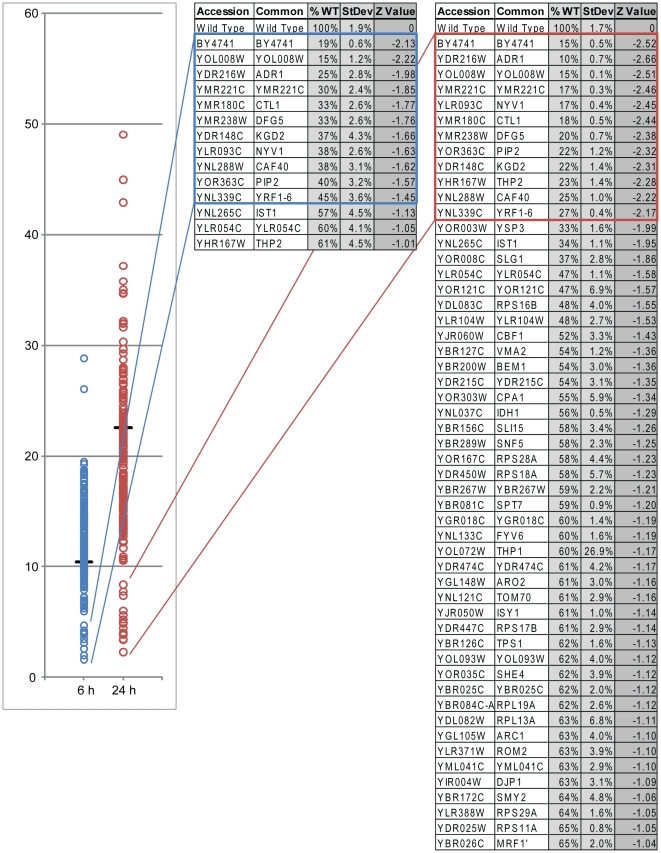
Flow cytometry analysis of candidate deletion strains. Plot of the log_10_ fluorescence of candidate deletion strains tested at 6 h and 24 h of oleate incubation. Deletion strains are indicated with open circles, while wild type is indicated by the line. The tables at the right show the flow cytometry analysis of the candidate genes with percentage of Pot1p-GFP fluorescence relative to wild type, the mean of the fluorescence values and the z value or standard score of the fluorescence at 6 h or 24 h respectively. Genes are shown that show decreases of Pot1p-GFP fluorescence that are at least 1SD from the wild type *POT1-GFP* strain. Genes boxed by blue or red indicate the naturally occurring separation of values shown in the plot at 6 h (blue) and 24 h (red). The remaining flow cytometry results are shown in Table S1-2. BY4741 is used as a non-fluorescent control strain.

At 6 hours post induction, 10 gene deletion mutants (N = 10) displayed perturbed expression of Pot1p-GFP (Figure 1 and Table S1-2). This group of gene deletions showed levels of Pot1p-GFP fluorescence that were more than 1 standard deviation (SD) below wild type levels, with a naturally occurring separation at a SD of 1.45 below wild type. Included in this group are two transcription factors known to regulate peroxisome biogenesis, Pip2p [11], [12], [13], [14] and Adr1p [11], [13], [14], [15], [16].

At the later stages of induction (24 h post induction), a natural clustering of 11 strains in which Pot1p-GFP levels were 2SD below wild type was observed (Figure 1 and Table S1-2). These strains include the transcription factors Pip2p and Adr1p, as well as additional nuclear and mitochondrial related proteins. A search of the respective annotations revealed that these proteins are of diverse localizations and functions. The gene products for the largest portion of this group show nuclear localization (Adr1p, Pip2p, Ctl1p, Thp2p, and Yrf1-6p), though deletions of mitochondrial (Coq10p, Ysp3p, and Kgd2p), and vacuolar (Nyv1p) proteins, as well as cytoplasmic proteins (Caf40p and Ist1p), also resulted in diminished expression of Pot1p-GFP (Figure 1B).

### Identification of Peroxisomal Matrix Protein Mislocalization Mutants

**Figure 2:**
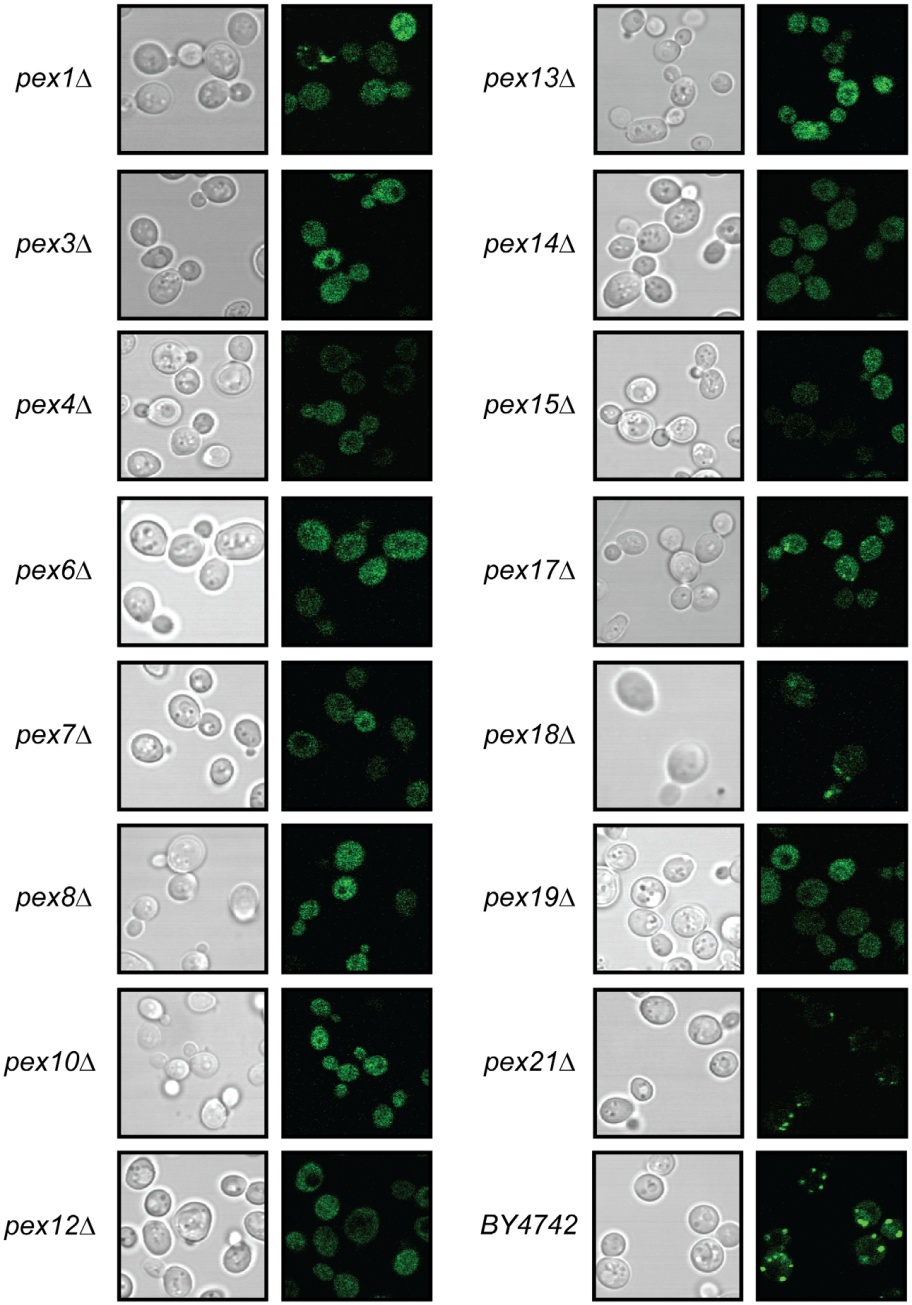
Identification of peroxisomal mutants. Bright field images are shown on the left panel and fluorescence images are shown on the right. Known peroxins show mislocalization of the Pot1p-GFP reporter when deleted. Partial mislocalization phenotypes are seen in *pex1*Δ, *pex18*Δ, and *pex21*Δ.

To complement expression data and to reveal genes required for peroxisome biogenesis *per se* the mutant library was also examined for the presence of morphologically normal peroxisomes using the Pot1p-GFP reporter and confocal microscopy. We present this imaging data as the Peroxisome Biogenesis Effectors Imaging Database, a resource for parties interested in both the functional genomics of peroxisomes and images analysis (http://PBEID.systemsbiology.net/). Immediately obvious in this screen were 18 strains in which the Pot1p-GFP signal was mislocalized. As expected, these included 14 previously identified pexes (Pex1p, Pex3p, Pex4p, Pex6p, Pex7p, Pex8p, Pex10p, Pex12p, Pex13p, Pex14p, Pex15p, Pex17p, Pex18p and Pex19p). While Pex18p and Pex21p have previously been demonstrated to be involved in localization of PTS2-bearing proteins, such as Pot1p, to the peroxisome [17], in this assay deletion of *PEX18* only partially mislocalized Pot1p-GFP and the deletion of *PEX21* showed normal peroxisomal localization of Pot1p-GFP (Figure 2).

Deletions of four additional genes, *YGL152C*, *YJL211C*, *OPT1*, and *CBS1*, led to mislocalization of Pot1p-GFP. *YGL152C* is a dubious open reading frame which partially overlaps with *PEX14*, which is likely the gene underlying the localization defect seen in the *ygl152c* deletion strain. *YJL211c* is likewise a dubious open reading frame that overlaps with *PEX2*, a previously characterized peroxisome-related locus whose product is known to form a complex with Pex10p and Pex12p which is suggested to act in the recycling of Pex5p during the peroxisomal protein import cycle [18], [19], [20], [21], [22]. *OPT1* and *YJL211c* are adjacent to, or overlap with, *PEX2* respectively, raising the possibility that the *opt1*Δ mislocalization defect is due to disruption of *PEX2*. Interestingly we found that a diploid *opt1*Δ/*opt1*Δ strain mislocalized Pot1p-GFP while *pex2*Δ/*pex2*Δ mutants localized Pot1p-GFP normally, (Figure S1A) suggesting that these strains are switched in the deletion library.

In order to establish whether *OPT1* or *PEX2* was the *bona fide* peroxin we cloned *OPT1* and *PEX2* into the yeast vector pRS316 and transformed putative deletion strains of *OPT1* and *PEX2*. We found that the pRS316 *PEX2* construct was able to complement the Pot1p-GFP import defect, demonstrating that the strain in the yeast deletion library position 119F12 is *pex2*Δ rather than *opt1*Δ while position 119G1 is *opt1*Δ rather than *pex2*Δ (Figure S1B).

The fourth novel gene necessary for Pot1p-GFP localization to peroxisomes is *CBS1*, which functions as a translational activator of cytochrome B mRNA [23]. Deletions of *CBS1* resulted in mislocalization of Pot1p-GFP. When we attempted to validate this phenotype, we found that the cells would partially recover normal signal over a period of several days, if maintained on a solid growth medium (Figure S2A). Deletions of *CBS1* have previously been reported to show an inability to grow on oleic acid as a sole carbon source [9], [10] and we observed the same phenotype (Figure S2B). This phenotype is suppressed over time, reproducibly coincident with the recovery of the Pot1p-GFP localization. We propose that *CBS1* is required for normal peroxisomal biogenesis, but that this mutant is readily suppressed, the mechanism of which remains unclear.

### Identification of Genes Regulating Peroxisomal Inheritance

**Figure 3:**
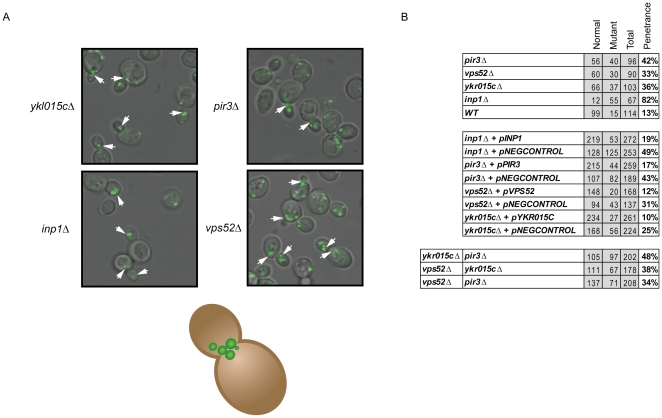
Vps52p, Pir3p and YKL015C are novel peroxisome inheritance factors. A. Strains lacking Inp1p, Vps52p, Pir3p or YKL015Cp accumulate peroxisomes near the bud neck or in daughter cells as indicated by the arrowheads. B. Summary of the effect of (i) deletions, (ii) complementation, and (iii) double deletions of *vps52Δ*, *pir3Δ* and *ykl015cΔ*.

Analysis of the confocal microscopy data identified a number of mutants that showed unusual distribution of peroxisomes, potentially reflecting defects in peroxisome inheritance to daughter cells (Table S1-1). The archetypal genes involved in peroxisomal inheritance are *INP1,* deletions of which fail to retain peroxisomes in the mother cell [24] and *INP2*, deletions of which fail to transmit peroxisomes to the daughter cells [25]. By single blind assessment we identified *inp1*Δ, and *pir3*Δ, *vps52*Δ and *ykr015c*Δ as defective in peroxisomal inheritance (Figure 3). Cells deleted for these genes were characterized by a tendency to cluster the peroxisomes at the bud necks or at sites of bud formation, and by a paucity of peroxisomes in mothers compared to newly formed buds (Figure 3A). Relatively subtle and variable phenotypes, such as that observed *inp2*Δ cells, in which cells fail to efficiently transmit peroxisomes to daughter cells [25] were not readily apparent in the initial visual screening but could be observed upon closer examination (data not shown). It is possible that there are additional such subtle defects that have gone undetected by this analysis.

We then investigated potential genetic interactions between the newly identified peroxisome inheritance regulators (*PIR3*, *VPS52* and *YKR015C*). Combinatorial deletions were done for *pir3*Δ, *vps52*Δ and *ykr015c*Δ, with no visible morphological evidence of additive effects for any of the deletion pairs (Figure 3B), suggesting that these gene products act independently of one another.

### Abnormal morphology

**Figure 4:**
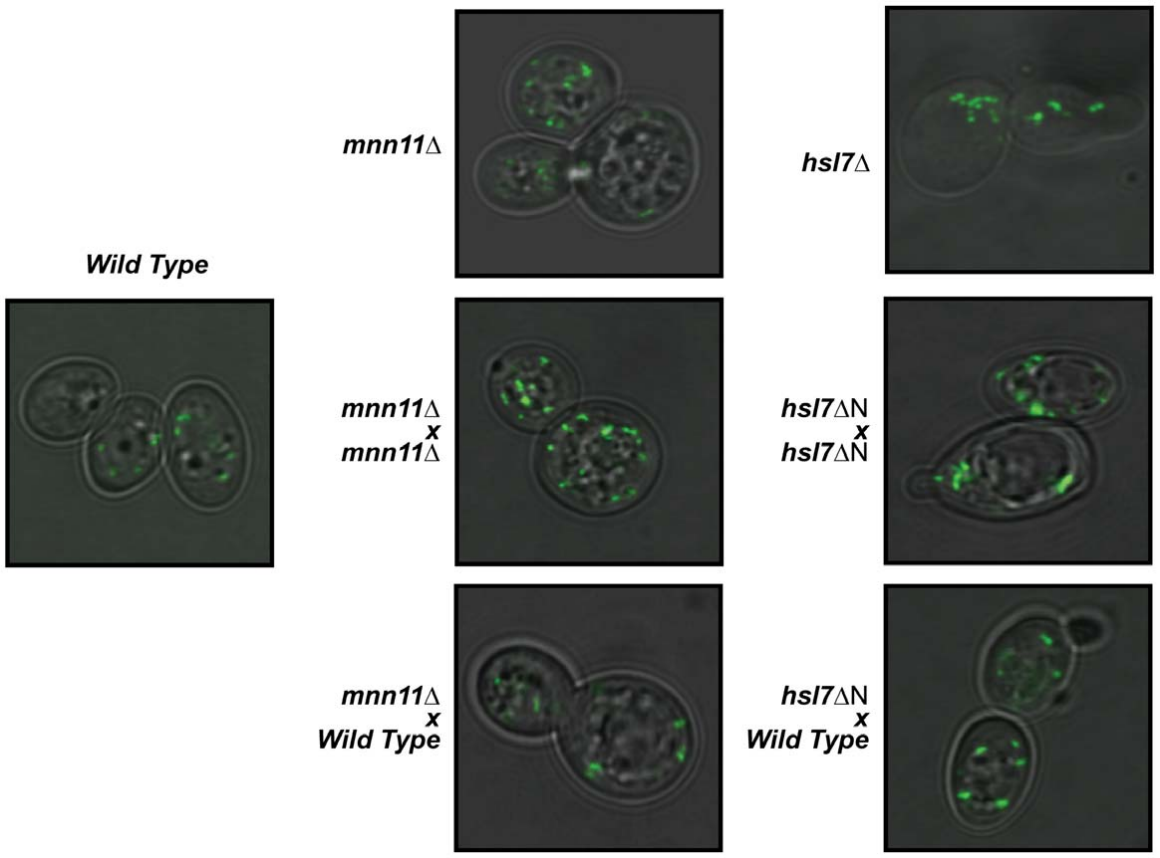
Mnn11p and Hsl7p are regulators of peroxisome morphology. Strains deleted for *mnn11* or the 5′ end of *hsl7* (*hsl7*ΔN generated by the *ybr134w* deletion) were backcrossed against an allelic deletion or a wild type strain (*BY4742*). An increased number of small peroxisomes are seen in cells deleted for *mnn11*, while *hsl7*ΔN lead to an increase in size or tendency for the peroxisomes to cluster. Both phenotypes are complemented by mating to the wild type strain.

The confocal microscopy analysis also identified several genes necessary for normal peroxisomal morphology - that is mutations resulting in smaller or larger peroxisomes compared to wild type. Deletion strains that were annotated as having large or small peroxisomes were tested by taking a series of high quality images that were then analyzed for peroxisomes using a novel image analysis method (Materials and Methods). Using this approach in combination with complementation analyses, we validated the phenotypes of two novel mutants, *MNN11* and *HSL7*, both of which are required for controlling peroxisome size (Figure 4). Deletions of *MNN11* resulted in smaller peroxisomes while deletions of *HSL7* resulted in both peroxisomal clustering and abnormal bud morphology. Longer time course microscopic analysis also identified known peroxisomal morphology regulators (Pex11p, Vps1p, and Dnm1p; data not shown).

Aberrant peroxisome morphology associated with *HSL7* was also observed in the *ybr134w*Δ strain, but this was attributable to *HSL7*, which overlaps with the dubious open reading frame annotated as *YBR134w*. To establish that this phenotype was linked to *HSL7*, the region was sequenced in various strains, and qPCR was used to evaluate expression. These experiments supported the annotation of *YBR134w* as a dubious ORF and revealed that *HSL7* was not deleted in the commercial library (see Materials and Methods).

### Meta analysis of peroxisome biogenesis factors

The deletion strains identified in this study were integrated with data sets generated by separate systems level analyses of regulators of peroxisome biogenesis [2], [26], or by the ability of *S*. *cerevisiae* to utilize fatty acids [9], [10]. In total, there were 372 effectors identified in at least one of the data sets (data not shown). Because peroxisome biogenesis effects within deletion strains can be variable upon validation [9], those genes that have been identified in at least two system wide analyses are included in Table S2, for a total of 211 validated regulators in all the datasets. Comparison of this data set with complementary datasets reveals that of 184 previously identified regulators of fatty acid utilization or peroxisome biogenesis, 143 (78%) were identified by this study. We also identified and validated an additional 26 regulators, bringing the total number of peroxisome biogenesis regulators identified by this study to 169. Table S2 thus represents the most comprehensive validated data set of effectors of peroxisome biogenesis across the high throughput studies related to peroxisomal function published to date.

Importantly, this analysis now facilitates the assignment of specific peroxisomal phenotypes to known regulators of peroxisome biogenesis or fatty acid utilization. These phenotypes include perturbations to peroxisomal morphology, including the size, number and distribution of peroxisomes, defects in localization of peroxisomal proteins, and perturbations to the robust expression of peroxisomal loci (represented in this study by decreased levels of Pot1p-GFP), likely through reduced transcription, translation or protein stability.

Two of the high throughput studies related to peroxisome biogenesis focused on the ability of deletion strains to utilize fatty acids as a sole carbon source (studies II and IV) [9], [10]. Of the 175 regulators identified in the fatty acid utilization studies, we identify a subset of regulators (8 genes (5%)), in which peroxisomes appear to be wild type with respect to Pot1p-GFP levels and peroxisomal morphology, that exclusively perturb the ability of *S. cerevisiae* to utilize fatty acids. While phenotypes were not obtained for 31 (NA in Table S2) of the regulators that appear in Table S2, we nonetheless expect that in general, genes that perturb fatty acid utilization also perturb peroxisome biogenesis.

**Figure 5:**
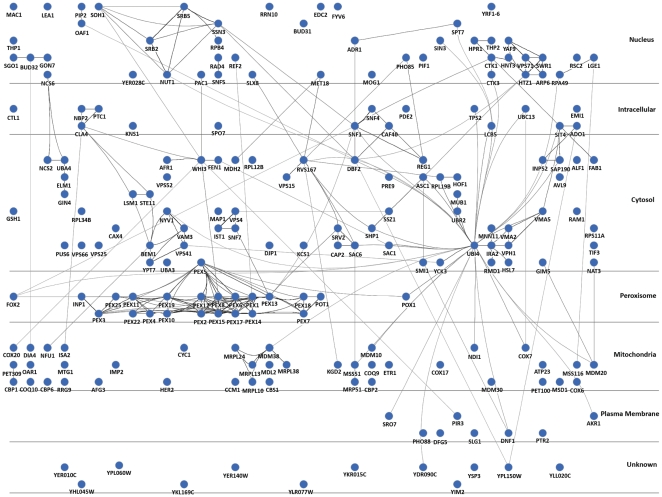
The cellular network regulating biogenesis of peroxisomes and the utilization of fatty acids. The circular nodes represent proteins identified in Table S2 and protein-protein interactions from the literature shown by edges between the nodes. For ease of display, protein localized to the vacuole, ER and Golgi are represented by the annotation “Cytosol”. Distinct functional modules within the nucleus, peroxisomal and mitchondrial localizations can be seen as well as the high connectivity of ubiquitin (UBI4).

Analysis of the associated GO annotations reveals the complexity of the peroxisome biogenesis program, involving components from multiple locations and involvement of a number of different processes, including regulation of chromatin reorganization, RNA polymerase II, vacuolar protein sorting and actin regulation (Figures 5 and S3). Significantly enriching GO annotations were identified for four key compartments or processes; peroxisomal organization regulators (p value: 4.68E-22), mitochondrial organization regulators (p value: 2.90E-06), cytoplasmic localization (p value: 9.38E-07) and serine/threonine kinases (p value: 6.52E-06). These and additional processes (for example mitochondrial ribosomes or ubiquitination), form regulatory modules of physically interacting proteins, as seen in Figure 5, regulating the biogenesis and morphology of peroxisomes.

## Discussion

We used a genomically integrated, GFP-tagged peroxisomal matrix enzyme to systematically analyze the effects of gene deletions on the peroxisome biogenesis program. In our approach, large amounts of both flow cytometry and three dimensional confocal data were captured for oleate-induced yeast cells, to identify deletion strains displaying inabilities to induce peroxisomes or aberrant peroxisomal morphology. These imaging data are available as a searchable database (PBEID) and can be downloaded at (http://PBEID.systemsbiology.net/). Through these methods, a number of candidate genes were identified with potentially biologically interesting phenotypes. The phenotypes identified included the mislocalization of Pot1p-GFP, low Pot1p-GFP fluorescence levels, abnormal transmission of peroxisomes to daughter cells, and abnormal peroxisomal size. These phenotypes were validated or excluded using high quality confocal imaging, low throughput flow cytometry analysis, complementation analysis, and informatics approaches.

Transport of proteins to the peroxisomal matrix depends upon peroxisomal targeting signals (PTS) of two types; Pex5p dependant type I signals (PTS1) [27] and Pex7p dependant type II signals (PTS2)[28]. In these assays, we used PTS2 dependant Pot1p fused to GFP as a marker for peroxisome biogenesis. Our analysis identified most of the known peroxins required for localization of peroxisomal matrix enzymes including Pex7p, whose gene product is the cytosolic transporter of PTS2 containing proteins. Through complementation studies, it was also ascertained that in the commercially available gene deletion library, what is annotated as an *OPT1* deletion is actually a *PEX2* deletion and what is annotated as the *PEX2* deletion is actually an *OPT1* deletion. *YJL211C* and *YGR152C* are annotated as dubious open reading frames and deletion of these ORFs disrupts *PEX2* and *PEX14* respectively, accounting for the mislocalization of Pot1p-GFP seen in these strains.

Four genes were identified whose protein products have effects on the inheritance of peroxisomes into daughter cells, including Inp1p [24], which was previously identified as being involved in this process, and three novel effectors of inheritance, Vps52p, Pir3p, and YKR015C. Inp1p is thought to connect peroxisomes to the mother cell cortex; in the absence of Inp1p and these newly identified genes, peroxisomes could be seen aggregating at the bud site or disproportionately partitioning into the newly forming daughter cells. *VPS52* is a regulator of actin with known genetic interactions with *ACT1*; mutations in *VPS52* can suppress temperature sensitive alleles of *ACT1* [29]. Peroxisomes are transported to daughter cells through a process that is thought to exclusively involve networks of actin upon which myosin motors act to reposition the organelle (for review see [30], [31]). Pir3p is required for cell wall organization and stability [32], while the function of YKR015C is unknown. How these proteins regulate the transmission of peroxisomes into daughter cells is currently unknown. The role of each of the newly identified inheritance factors does not appear to be dependent on the other newly identified factors, as pair wise deletions of these genes did not amplify the inheritance defect. Taken together, these data indicate that additional factors are involved in the inheritance process, including additional regulators of actin organization.

Our analysis of the data from this study and the extant literature, presents an overview of the genes, cellular components, and processes that govern peroxisome biogenesis. The protein products of the genes identified in this analysis localize to a number of different sites and are involved in a number of different processes. We find groups of proteins, including those which physically interact and form putative regulatory modules, regulating cytoskeleton components, ubiquitination, mRNA processing, RNA polymerase I and II, and signal transduction events. These processes are presumably distributed to a number of sites throughout the cell including the nucleus, ER, mitochondria, vacuole, and cytosol, in addition to the peroxisome itself (Figure 5, Figure S3). These proteins, thus, form site specific networks of interactions, ultimately generating a “supernetwork” of protein interactions within the cell, which governs the peroxisome biogenesis program (Figure 5, Figure S3, and Table S2).

Evidence for dynamic interplay between peroxisomes and the ER, the mitochondria and the vacuole has been accumulating over the last few years. Our initial characterization of the proteome of the peroxisome revealed a number of ER resident, vacuolar and mitochondrial components that specifically enriched with and were localized to the peroxisome during oleate incubation of *S. cerevisiae* [7], [8]. Through this present study, we characterize the influence of these organelles on the biogenesis of peroxisomes and can begin to infer normal roles for proteins on peroxisome morphology on a genome-wide scale. Of particular note is the number of mitochondrial proteins whose absence leads to a morphological defect in peroxisomes. Increasingly, the interconnectivity of peroxisomes and mitochondria is being recognized. These organelles share metabolic enzymes, division components, and very recently a novel vesicular mitochondrion-to-peroxisome pathway has been characterized [33], the physiological function of which is not understood. This study shows the dependence of peroxisome biogenesis on proteins attributed to mitochondrial function, demonstrated by the phenotypes detected in the absence of these proteins. These phenotypes varied from a mislocalization phenotype (*CBS1*), to reductions in peroxisomal numbers (Table S2), to severe reductions of expression of the Pot1p-GFP reporter (*KGD2*, *COQ10*; see Figure 1 and Table S2).

In our previous analysis of signaling regulators, in particular the regulators of reversible phosphorylation, we found that specific subsets of molecules regulate the ability of the cell to transition from a glucose repressed to a depressed state, then into a fatty acid-induced state where peroxisomal size and number increase. This study reinforces the role of key regulators such as *SNF4* and *PHO85* in peroxisome biogenesis and, as the strains in this study are a hybrid of *BY4741*, demonstrates that the peroxisomal phenotypes hold true in genetically different backgrounds. We note however, the strains in this study are derived from a cross between two non-isogenic parental strains. The genetic background of these hybrid strains could influence the phenotype; however the strains used here are derived from standard laboratory strains. Nonetheless, detailed examinations of the genotype-phenotype relationships are important to reveal complex genetic influences not apparent in this survey. Furthermore, we now expand our analysis of signaling regulators in peroxisome biogenesis to ubiquitin regulation. In Table S2, a number of ubiquitin regulators of peroxisome biogenesis are identified (*SHP1*, *UBR2*, *MUB1*, *UBI4*, and *UBA4*) and our analysis identifies yet another ubiquitin regulator, *UBC13*. Interestingly, several peroxisomal targeting signal receptors are targets of mono and poly ubiquitination (Pex5p [34], [35], [36], Pex18p, Pex21p [37]) (for a review see [38]). The ubiquitination of Pex5p is required for its recycling [35], [39] and several peroxins (Pex4p [40], [41], Pex10p [42], Pex2p and Pex12p [43]) resemble ubiquitin conjugating enzymes.

Significant inroads have been made into our understanding of the regulation of the transcriptional architecture [6], [44], chromatin dynamics and nucleosome structure [3], signaling cascades [2] and proteomics [7], [8] that govern the peroxisome biogenesis program. This study provides not only a genome-wide view of the effect each gene deletion has on peroxisome morphology, but also provides an extensive framework from which to further query the complex array of processes that regulate the response of cells to fatty acids and the biogenesis of peroxisomes.

## Materials and Methods

### Library construction and cell culture

To construct strains expressing the Pot1p-GFP chimera, the *POT1* gene was tagged at its 3′-end through PCR-based homologous recombination in-frame with the sequence encoding *A. victoria GFP* [45] using *HIS5* as an auxotrophic marker. The *HIS5* cassette was exchanged for a natR cassette by homologous recombination and the resulting *POT1*-*GFP natR* cassette was then amplified by PCR and transformed in the query strain *Y8205* (provided by C.Boone). The mating strategy to create the library of deletion strains with *POT1*-*GFP* integrated was performed as described by Tong et al [46], [47]. The resulting deletion *POT1*-*GFP* library was stored in 96 well plates in YEPD and 15% glycerol.

The yeast deletion library is comprised of 4,827 individual deletion strains, of 52 which are duplicates, leaving a total of 4775 unique deletion strains. After initial construction of the library, there were 256 strains which failed to survive the mating and selection process. The library was also tested for mating type [48], eliminating another 457 strains that were either the α-mating type or diploid cells. After elimination of strains for which no data could be acquired (failure to grow or lacking the *POT1-GFP* integration), there were 4049 strains remaining. This study thus represents an analysis of 85% of the deletion strain library.

### Flow Cytometry

For initial induction profiles (16 hours), deletion strains were grown in YPBD (1% yeast extract, 2% peptone, 2% glucose) to mid-log phase in 96-well deep-well plates, pelleted, washed with water, resuspended in the same volume of YPBO (0.3% yeast extract, 0.5% peptone, 0.5% potassium phosphate buffer, pH 6.0, 0.5% Tween 40, 0.2% oleic acid) and incubated with shaking for 16 h at 30°C. After incubation, the cells were washed and fixed with formaldehyde (3.7% final) for 30 min, washed with dH_2_O and analyzed by flow cytometry using a BD Biosciences FACSCalibur with the following parameters: forward scatter (FSC) - E0 haploid, linear scale; side scatter (SSC) -520V linear scale; fluorescence (FL1) - 490V logarithmic scale. Cells were loaded onto the FACSCalibur using the BD Biosciences high throughput sampler (HTS). The HTS was run in standard mode using a 96-well flat bottomed plate and was set to sample 10 µl at a rate of 2 µl/sec.

Analysis of the flow cytometry data was performed by a regression model that reduces the coefficient of variation by removing cell size and granularity effects (Knijnenburg et al, in preparation). Plate to plate variation was normalized using quantile normalization [49], [50] and the fluorescence reported as a standard score (Z-score) relative to the population mean and standard deviation.

Resulting candidate strains were cultured in 5 ml of YPD overnight at 30°C, washed with dH2O, and incubated in 2 ml YPBO in 15 ml culture tubes 30°C for 6 and 24 h post induction, and analyzed by flow cytometry as described above.

### Confocal fluorescence microscopy

To prepare the cells for visualization by confocal microscopy, strains were cultured at 30°C in YPBD overnight, washed with dH_2_O, and incubated in YPBO in 96 well Beckman plates, shaking at 30°C for 6 hours. After incubation the cells were washed and fixed with formaldehyde (3.7% final) for 30 min, washed with dH_2_O and imaged by confocal microscopy. For high quality images cells were treated as described for flow cytometry of candidate genes.

Stacks of twenty images along the z-axis were captured with a Plan-Apochromat 100.0×1.40 Oil UV objective on an Axiovert 200 inverted microscope equipped with a LSM 510 META confocal scanner (Carl Zeiss). GFP was excited with a 488-nm laser, and its emission collected using a 505-nm long-pass filter. Images were captured at 23°C with the microscope pinhole adjusted to 0.5 Airy units.

### Complementation Assays

To address the true nature of the *pex2* and *opt1* deletion strains, segments of genomic DNA containing the ORF of interest, and 500 bp of untranslated region 5′ and 3′ of the ORF were amplified by PCR, and cloned into the yeast vector pRS316 [51]. Plasmid transformed yeast were grown in complete media - URA (0.67% Yeast Nitrogen Base, 5% Ammonium Sulfate, supplemented with CSM-URA (Bio 101)).

For complementation of deletion strains, plasmids containing the ORF of interest from the Open Biosystems Genomic Tiling Collection were used (Open Biosystems #YSC4613). Plasmid DNAs were isolated from bacteria and transformed into yeast deletion strains of interest. Transformed strains were grown in complete media - LEU. For the *vps52*Δ strain, no clone was available in the open biosystems library. The ORF, with 500 bp of UTR 5′ and 3′ of the ORF, was amplified by PCR, and cloned into the yeast vector pRS315 [51].

Since *YBR134W* and *YBR133C* ORFs overlap, the regions including *YBR133C*, *YBR134W* and *YBR135W* were sequenced in *ybr134*Δ, *ybr133*Δ and *BY4742*, and the expression from these ORFs was evaluated in the same strains by quantitative RT-PCR. These experiments determined (i) that transcription from *YBR134W* is negligible, supporting the notion that it is a dubious ORF (ii) that deletion of *ybr134w*Δ in the commercial library disrupted the 5′ end of *YBR133C*, leading to poor expression of this *YBR133C* in the *ybr134w* strain, and (iii) the strain from the commercial (Resgen) deletion library reported to be a deletion of *ybr133c* was incorrect; it contained a wild type copy of *YBR133C*. Therefore new strains carrying deletions of *hsl7* (*ybr133c*) and *cks1* (*ybr135w*) were constructed using a hygromycin cassette with ends homologous to the 5′ and 3′ regions of the respective genes as described previously [52]. Correct integration was confirmed by unambiguous PCR analysis. Cells were induced with YPBO over an 8 hour time course, formaldehyde fixed and imaged as described above.

### Cytoscape Analysis

Known physical interactions between proteins were downloaded from the *Saccharomyces* Genome Database (February 03, 2010). The network was then visualized using the Cerebral plugin in Cytoscape (version 2.6.2), arranging the nodes in the network by subcellular localization. Subcellular localization terms used were ‘Nucleus’, ‘Intracellular’ for those proteins which localize to both the nucleus and cytosol, ‘Cytosol’ for those proteins which localize not only to the cytosol but also to organelles, for example the ER, vacuole and Golgi (see Figure S3), ‘Peroxisome’, ‘Mitochondria’ and finally ‘Unknown’.

### Image Analysis

We developed an image analysis method that can find the volumes of the peroxisomes from the image stacks. This method can be applied using high quality images in which the cells are, for example, immobilized using an agarose slide, and was applied here for selected strains. Cells were induced and fixed as described above, and then laid onto an agarose bed on a microscope slide.

Cell segmentation is done with the same K-means clustering method as described previously [53], [54], but peroxisome segmentation is done for each image slice in the image stack in order to obtain peroxisome volumes. The method first uses a low-pass filter that preserves only peroxisome-like features and ignores noisy high-intensity areas. Thresholding is then performed to define peroxisome areas in each slice. Combining the thresholded slices back into an image stack allows us to readily obtain the peroxisome volumes.

### Fatty Acid Utilization Plate Assays

Cells were tested for the ability to utilize fatty acid by growth on oleate-containing media as described previously [2], [10].

## Supporting Information

Figure S1 The deletion strain at position 119F12 in the Yeast deletion library is *pex2*Δ. At the top is a schematic of genomic structure of *PEX2* and *OPT1*. The allelism of *pex2*Δ was demonstrated in two ways. On the left, the strains from the designated library positions (Plate 119, Row F, column 12 or Row G, column 1) were crossed with the GFP containing strains from equivalent library positions. Only the 119F12×119F12 cross (119F12 is designated as *opt1*Δ) yields the mislocalization phenotype. On the right, *PEX2* was expressed in the putative *opt1*Δ deletion strain and rescued the mislocalization phenotype, while neither *OPT1* expression nor empty plasmid was able to do so. We therefore conclude that 119F12 is actually a deletion of *PEX2*.
(3.74 MB TIF)

Figure S2 Deletion of *CBS1* leads to a mislocalization phenotype that is susceptible to accumulation of suppressors. A. Mislocalization of the Pot1p-GFP reporter. At day 5 after recovery from frozen stock, most cells show a mislocalization phenotype while after 46 days post recovery, most cells show localization of the reporter to peroxisomes. B. The ability to utilize fatty acids coincides with the ability to localize the Pot1p-GFP reporter to peroxisomes. In the top panel are shown strains deleted for *PEX3* or *PEX7* (the PTS2 and thus Pot1p transporter). In the bottoms strains are shown the *CBS1* deletion strains at day 5 (*cbs1*Δ^1^) and day 46 (*cbs1*Δ^2^). The strain at Day 46 is now a mixed population of cells that are unable, and cells that are able to utilize myristic acid as a sole carbon source.
(1.44 MB TIF)

Figure S3 Peroxisome biogenesis is a complex process involving a number of organelles and processes. The nucleus is shown in red, endoplasmic reticulum in blue, Golgi apparatus in purple, ribosome in deep purple, vacuole in white, mitochondrion in brown, cytoplasm in grey and peroxisome in green. Note that intracellular refers to genes with annotations to both the cytoplasm and nucleus.
(2.66 MB TIF)

Table S1 Flow Cytometry and Image Analysis. S1-1. Genome Wide Flow Cytometry and Imaging Analysis of Peroxisome Biogenesis Effectors. S1-2. Candidate Testing - Flow Cytometry. S1-3. Deletion Strains Not Studied.
(0.63 MB XLS)

Table S2 Regulators of Peroxisome Biogenesis. Deletion strains that show a perturbation in peroxisome biogenesis or a reduced capacity to utilize fatty acids are shown. The studies used in construction of this table are indicated by columns I through V. Column I.a. refers to the initial flow cytometry analysis. Column I.b. refers to the manually curated imaging data (phenotype key is given in Table S1-1) as well as the independent validations. Column II refers to data from [10], III from [2], IV from [9], and V from [23]. A gene deletion strain had to be identified in at least two of the global studies for inclusion, with the number of studies in which the gene was identified shown by the column marked ‘#’, with the exception of the novel regulators that were identified and independently validated by this study, indicated in the column ‘*’. Localization and Primary function in the penultimate and ultimate columns were ascertained from the literature and the SGD database.
(0.28 MB XLS)
