# Supplementary figures and images for: Genome-Wide Analysis of Effectors of Peroxisome Biogenesis
Source: PLoS One. 2010 Aug 4;5(8):e11953. doi: 10.1371/journal.pone.0011953 (PMC2915925; doi:10.1371/journal.pone.0011953)

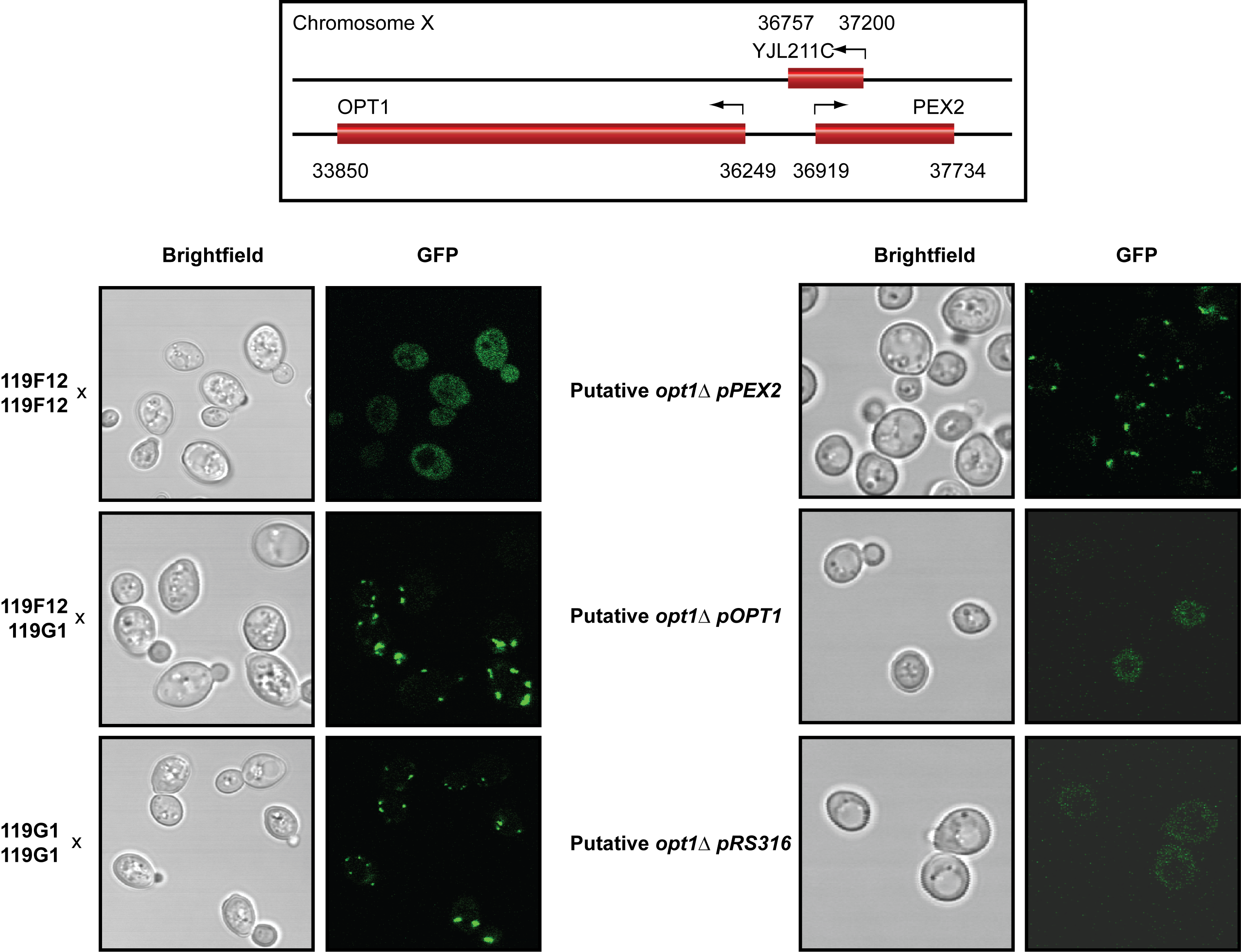

Supplement: Figure S1 — The deletion strain at position 119F12 in the Yeast deletion library is pex2Δ. At the top is a schematic of genomic structure of PEX2 and OPT1. The allelism of pex2Δ was demonstrated in two ways. On the left, the strains from the designated library positions (Plate 119, Row F, column 12 or Row G, column 1) were crossed with the GFP containing strains from equivalent library positions. Only the 119F12×119F12 cross (119F12 is designated as opt1Δ) yields the mislocalization phenotype. On the right, PEX2 was expressed in the putative opt1Δ deletion strain and rescued the mislocalization phenotype, while neither OPT1 expression nor empty plasmid was able to do so. We therefore conclude that 119F12 is actually a deletion of PEX2. (3.74 MB TIF) [file pone.0011953.s001.tif]

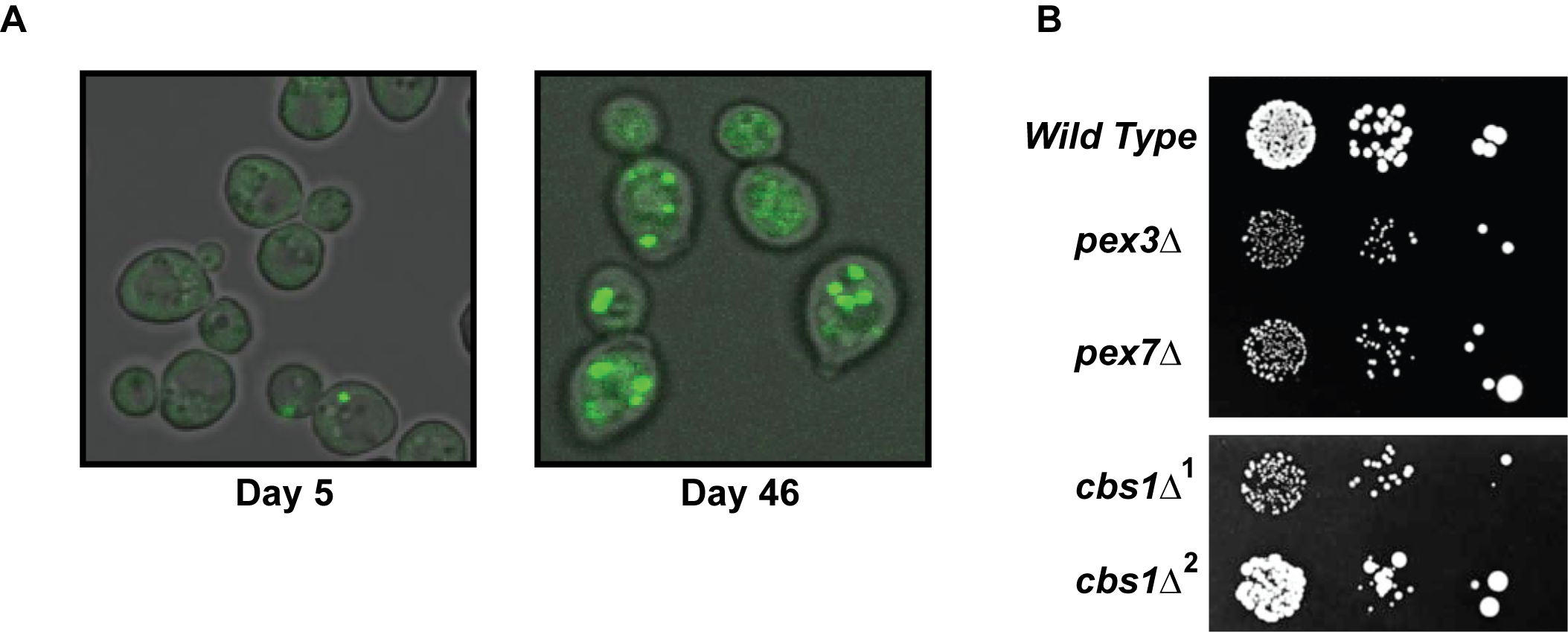

Supplement: Figure S2 — Deletion of CBS1 leads to a mislocalization phenotype that is susceptible to accumulation of suppressors. A. Mislocalization of the Pot1p-GFP reporter. At day 5 after recovery from frozen stock, most cells show a mislocalization phenotype while after 46 days post recovery, most cells show localization of the reporter to peroxisomes. B. The ability to utilize fatty acids coincides with the ability to localize the Pot1p-GFP reporter to peroxisomes. In the top panel are shown strains deleted for PEX3 or PEX7 (the PTS2 and thus Pot1p transporter). In the bottoms strains are shown the CBS1 deletion strains at day 5 (cbs1Δ1) and day 46 (cbs1Δ2). The strain at Day 46 is now a mixed population of cells that are unable, and cells that are able to utilize myristic acid as a sole carbon source. (1.44 MB TIF) [file pone.0011953.s002.tif]

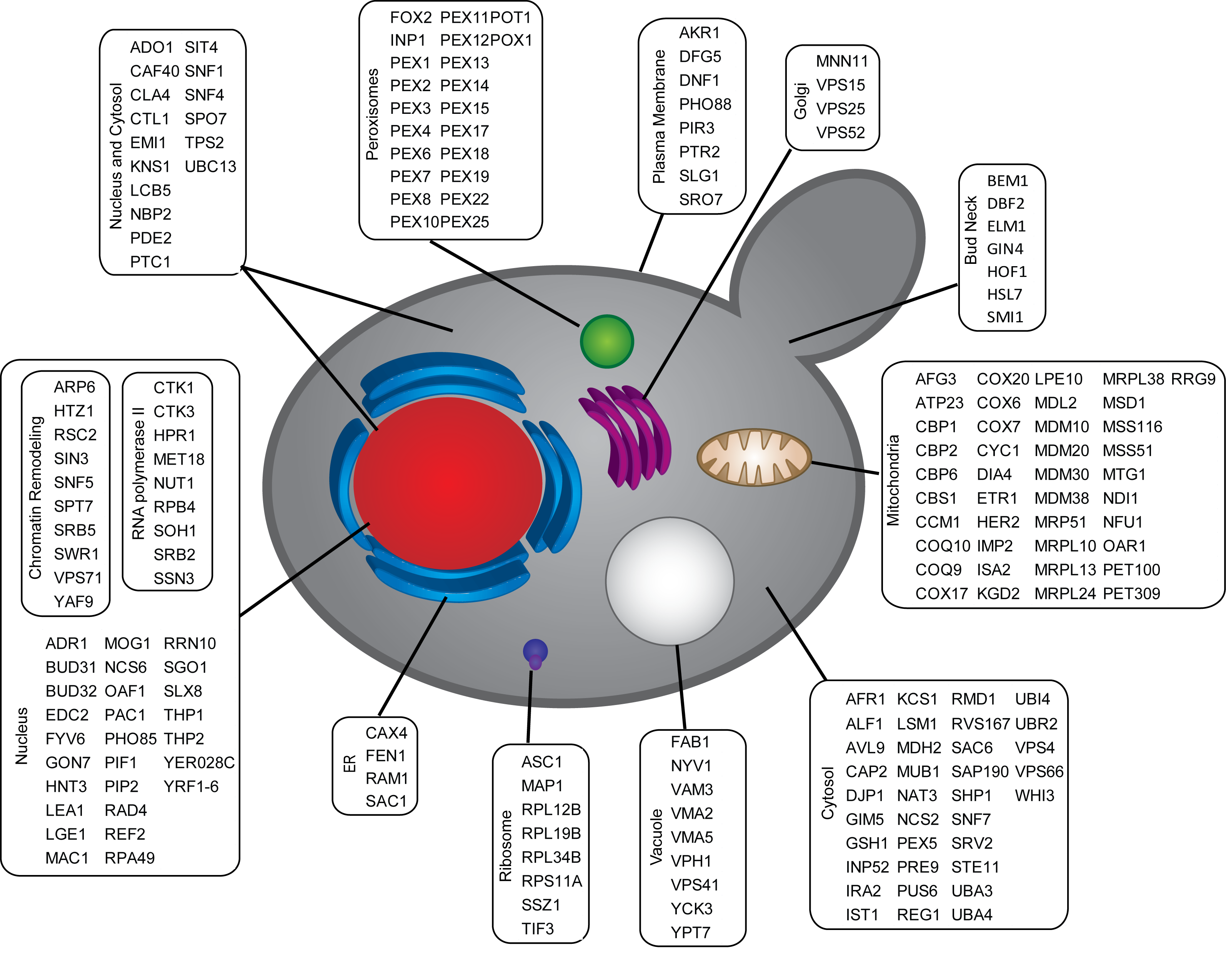

Supplement: Figure S3 — Peroxisome biogenesis is a complex process involving a number of organelles and processes. The nucleus is shown in red, endoplasmic reticulum in blue, Golgi apparatus in purple, ribosome in deep purple, vacuole in white, mitochondrion in brown, cytoplasm in grey and peroxisome in green. Note that intracellular refers to genes with annotations to both the cytoplasm and nucleus. (2.66 MB TIF) [file pone.0011953.s003.tif]
